# Trapping Elusive Cats: Using Intensive Camera Trapping to Estimate the Density of a Rare African Felid

**DOI:** 10.1371/journal.pone.0142508

**Published:** 2015-12-23

**Authors:** Eléanor Brassine, Daniel Parker

**Affiliations:** Wildlife and Reserve Management Research Group, Department of Zoology and Entomology, Rhodes University, Grahamstown, South Africa; INRA-UPMC, FRANCE

## Abstract

Camera trapping studies have become increasingly popular to produce population estimates of individually recognisable mammals. Yet, monitoring techniques for rare species which occur at extremely low densities are lacking. Additionally, species which have unpredictable movements may make obtaining reliable population estimates challenging due to low detectability. Our study explores the effectiveness of intensive camera trapping for estimating cheetah (*Acinonyx jubatus*) numbers. Using both a more traditional, systematic grid approach and pre-determined, targeted sites for camera placement, the cheetah population of the Northern Tuli Game Reserve, Botswana was sampled between December 2012 and October 2013. Placement of cameras in a regular grid pattern yielded very few (n = 9) cheetah images and these were insufficient to estimate cheetah density. However, pre-selected cheetah scent-marking posts provided 53 images of seven adult cheetahs (0.61 ± 0.18 cheetahs/100km²). While increasing the length of the camera trapping survey from 90 to 130 days increased the total number of cheetah images obtained (from 53 to 200), no new individuals were recorded and the estimated population density remained stable. Thus, our study demonstrates that targeted camera placement (irrespective of survey duration) is necessary for reliably assessing cheetah densities where populations are naturally very low or dominated by transient individuals. Significantly our approach can easily be applied to other rare predator species.

## Introduction

Many large African carnivores have disappeared from their historical ranges due to habitat fragmentation, prey depletion and direct persecution, and their persistence relies mostly on the success of adequate research and conservation strategies which include good management policy and public support [1]. To implement conservation actions effectively it is essential to have a sound understanding of the status and threats faced by resident carnivore populations and to identify research priorities [2]. The ability to reliably monitor large carnivore populations is key to adequately assessing the status and viability of populations [3], yet obtaining reliable estimates of large carnivore populations is challenging due to their expansive use of space, secretive nature and naturally low population densities [4].

Remotely-triggered camera trapping has become increasingly popular for monitoring rare, cryptic mammals as it is relatively inexpensive and non-invasive [2, 5]. Significantly, camera trapping can be successfully used to systematically survey individually identifiable big cats [6]. Individuals can be identified by unique natural markings such as spot or stripe patterns, which allow for population estimates to be calculated by way of capture-recapture methods [7].

The probability of detection in camera trapping surveys is a fundamental aspect that needs to be carefully considered in order to obtain robust estimates of population size [4]. A large enough sample size relies on the capture probability of the species being studied [7], which depends on a number of variables, such as survey design, habitat type, and most crucially on the behaviour and density of the target species [5, 6]. Additionally, it is essential that individuals of the target species can be reliably distinguished from each other throughout the study [8] and image quality and trap placement are therefore factors which must be carefully considered [9].

To have estimates as close to actual numbers as possible (and recognising that these fluctuate temporally) it is important to minimise bias, and this is achieved in the design of the survey [4]. An even sampling effort across the landscape and minimum survey length is, therefore, favourable [10]. However, the design itself relies heavily on the biogeographic characteristics of the species and the objective of the survey. Estimating abundances and densities of rare species requires more effort as detection rates are lower. In addition, certain sampling methods may only be effective for particular species; this is mostly due to habitat characteristics that strongly influence animal movements and therefore rates of encounter [4, 9]. For example, species that are found in dense vegetation may be forced to move along natural trails; so targeted placement of camera traps on these well-defined paths may increase photographic captures [4, 10–12]. However, it may be more difficult to predict the movements of species which occur in more open landscapes or species that do not make use of trails.

Cheetahs (*Acinonyx jubatus*) are individually recognizable, occur at very low densities, and are elusive, cryptic, and highly mobile [13–16]. Cheetahs sometimes aggregate at small-scale, local transient hotspots (e.g. in areas with high prey densities and low apex predator densities), and these may be inadvertently extrapolated to infer large-scale high cheetah density [16, 17]. Additionally, the large home ranges of cheetahs may give the false impression of high cheetah numbers due to repeat sightings of the same individual(s) over large areas [18–20]. These aspects of cheetah ecology make it extremely difficult to effectively monitor their populations. However, given the critical conservation status of cheetahs and other large carnivore species [21], it is important to have reliable population estimates to assess population trends and to adequately evaluate the success of conservation efforts [22].

In this study we explore the effectiveness of two camera trapping approaches (systematic vs. targeted camera placement) to produce population estimates of a free-roaming cheetah population in eastern Botswana. The influence of camera placement on cheetah capture rates and the effect of survey duration on sample sizes and the resulting population estimates were also assessed. Further, we provide recommendations for the design of camera trapping surveys for other rare large carnivore species with similarly low detection probabilities.

## Methods and Materials

The study was conducted under a research permit issued to the Northern Tuli Cheetah Project by the Wildlife Department and National Parks of Botswana. The project had the full support of the Northern Tuli Game Reserve and was approved by the Rhodes University Ethical Standards Committee (Ethical clearance number: ZOOL-03-2012).

### Study area

The study was undertaken in the Northern Tuli Game Reserve (NOTUGRE), a private game reserve situated in the eastern corner of Botswana. NOTUGRE is naturally delineated by the Shashe River in the east and the Limpopo River in the south. The former is the border between Botswana and Zimbabwe and the latter, the border between Botswana and South Africa. The game fences of NOTUGRE are limited to the western and the southern boundaries and do not restrict the movement of large carnivores; cheetahs, lions (*Panthera leo*), leopards (*Panthera pardus*), spotted hyenas (*Crocuta crocuta*) and African wild dogs (*Lycaon pictus*) frequently move across these fences [23].

NOTUGRE was established by multiple landowners in 1986 [24]. It consists of 36 individual properties and encompasses an area of 728km². These individual properties are used for commercial ecotourism or private holiday purposes [24]. One of the properties has some livestock, including goats (*Capra hircus*) and cattle (*Bus taurus*), and a citrus orchard which are enclosed within a game fence. There are also two non-member properties where the land is used for crop and pastoral (goats and cattle) farming. The camera trapping survey area was situated in the centre of the reserve and covered approximately 240 km². The location was chosen for practical purposes and also to avoid the edges of the reserve where the theft of cameras may have been a problem.

The climate of NOTUGRE can be described as semi-arid and sub-tropical with temperatures fluctuating between -5°C and 42°C [25]. Temperatures peak during December and January, and reach their minimum during June, July and August. Rainfall is low and unpredictable and the majority falls in the summer months between November and March, usually induced by convectional movements. The average annual precipitation is 386.5mm (for the years 1996–2013). NOTUGRE has an average elevation of about 600m.a.s.l and falls within the Mopane Bioregion of the Savannah biome, classified as arid, base rich savannah [26]. The vegetation can be broadly classified as Mopane Veld, but is also made up of a wide variety of other smaller habitats [25].

Historical accounts of cheetahs in NOTUGRE are scarce. Between 1966 and 1971 only two sightings of two cheetahs were recorded and there were reports of a number (number unspecified) of cheetahs poached and found at a trading store south of the Motloutse River [27]. In 1974, Lind [28] described the cheetah as rare and only seen sporadically in NOTUGRE. He gives a few accounts of cheetah sightings and broadly estimates the population to have a total of seven individuals of unspecified ages in 1972 and nine individuals by the end of 1973. Numbers were estimated based on total number of sightings and not individual recognition. Lind [28] described the species as at risk of being locally extinct and recommended strict protection by keeping disturbance to a minimum. Prior to our study, there had not been any formal assessment of the NOTUGRE cheetah population.

Two camera-trapping surveys were carried out using two different trapping arrays. The first survey followed the more traditional approach of having camera traps set out uniformly over the landscape [7]. The second survey had camera traps placed at sites presumed to increase the probability of capturing cheetahs (targeted placement), resulting in an irregular pattern of trap locations across the study area [29].

### Regular trap configuration

**Fig 1:**
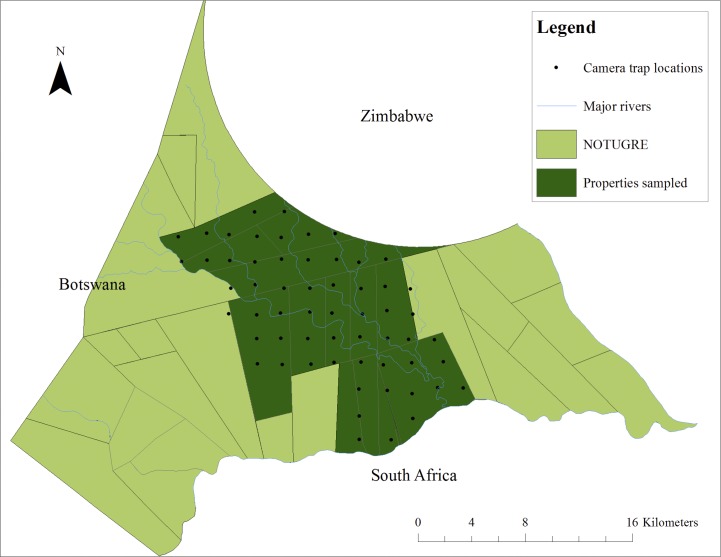
The locations of camera traps (n = 60) for the first survey using a systematic grid method. Solid blue lines symbolise the major rivers of the area and solid grey lines the boundaries of properties. The green polygons illustrate the properties included in the study area. The camera trap placements are marked as black dots and were chosen within 200m of the predetermined random points (ArcMap 10; projected: Transverse-Mercator, spheroid W GS84, central meridian 29; map units: meters).

Twenty Cuddeback Attack (Non Typical, Inc., Green Bay, WI, USA) camera traps were used at 60 locations within NOTUGRE (Fig 1). A regular trap configuration technique was used [7, 8, 30, 31], whereby predetermined points are distributed systematically in a grid although the actual placement of the camera is non-random, usually on a nearby animal path to increase the chances of capturing animals moving in the area. This approach to distributing camera traps in the study area ensures an even sampling effort across the landscape and assumes an equal detection probability for all individuals, reducing sampling biases from spatial variation in capture probabilities [10, 32]. Based on cheetah movements observed in the study area, a grid with equally spaced points at 3.7 km intervals was placed over a map of the surveyed area using ArcMap 10 (ESRI, Redlands, CA, USA). These predetermined points represented random camera trap placements, but actual camera traps were set within 200 m (mean distance and standard deviation = 164 ± 94 m) of the predetermined points, and thus had a tolerance of 4.4 ± 2.5%. Camera traps were placed within this buffer zone at sites presumed to maximise the likelihood of photographing a moving animal, preferably on animal paths [33]. The spacing between camera traps was based on the movements of a resident adult female cheetah with sub-adult cubs which was collared for research purposes [9, 34]. The daily distance travelled was calculated by adding the distance between consecutive locations within a day [35]. This average daily distance moved (3.7 km) was used to calculate the minimum distance between camera trap sites to ensure that no cheetah would go undetected [9, 10]. In other words, no cheetah has a capture probability of zero.

Using two opposing cameras per station is preferable to capture images of both sides of the animal for individual identification and to increase the detection rate [9, 36]. However, only one camera was used per station in our study so that more camera traps could be deployed over a larger area, thereby increasing the number of independent locations and maximising the chances of detecting cheetahs [32]. When surveying rare or sparse species it is best to sample broadly across the study area as this increases the likelihood of captures [32].

Typically, camera-trapping surveys are conducted over a short period to ensure demographic closure [9, 37]. Thus, the survey was carried out over a 90 day period during the hot/wet season (December–March 2013). Due to the large size of the survey area (±240 km²) and the limited number of cameras (*n* = 20), the Adjacent Block method [9, 10] was implemented to ensure that the whole sampling area was covered. The sampled area was divided into three sections and each section was sampled sequentially for approximately 30 continuous days [9]. The total number of days that cameras were active is the duration of the survey, with each day (24-h period) defined as a sampling occasion, starting at 12h00 and ending at 11h59 [7], when cheetahs are believed to be least active [38].

The cameras were set to take high quality (5MP) images and the strobe flash range was set at 30 feet (9.14 m). This was occasionally reduced to 10 (3.04 m) or 20 feet (6.09 m) when an animal was likely to come closer to the camera so as to reduce the risk of overexposed images. The cameras used four D-cell batteries, a 4GB SD card and a passive infrared sensor to detect heat and motion. The cameras were housed in steel protective casings and fastened to trees. Cameras were secured at approximately 0.3 m above the ground and were active 24h/day with a 1 minute delay between consecutive photographs to minimize unnecessary captures of gregarious, non-target species. The cameras were inspected, on average, every 15 days to replace batteries and memory cards and to ensure that they were operating normally. No baits were used at camera trap stations to prevent heterogeneous capture probabilities [32]. However, no effort was made to conceal human scent.

The number of active days, or trap-days, was calculated for each station. Every day that a camera was active was deemed one active day. If cameras malfunctioned, had technical problems (such as no flash triggered at night or flat batteries), or were damaged by elephants (*Loxodonta africana*) or flooding, those days were excluded from the data analyses. Thus, active days included only problem-free days.

### Non-random configuration using scent-marking posts

**Fig 2:**
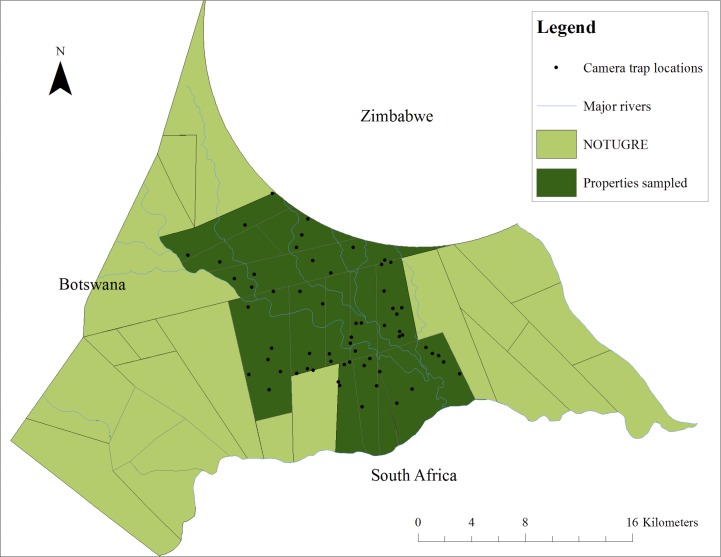
Camera trap locations (*n* = 60) at identified scent-marking posts for the second camera trap survey. (ArcMap 10; projected: Transverse-Mercator, spheroid WGS84, central meridian 29; map units: meters).

The second camera trapping survey used known cheetah scent-marking posts for camera trap locations [14, 15; 29]. Field guides working in NOTUGRE had observed cheetahs using scent-marking posts and, with their assistance, a total of 104 such sites were identified and mapped as potential trapping locations. A proximity test was run in ArcMap 10 to calculate distances between all scent-marking posts and data were cleaned; effectively removing scent-marking posts that were within 250 m of other scent-marking posts. Where more than one scent-marking post lay within a selected area, the site with the most recent signs of cheetah activity (presence of scats, urine spray, and tracks) and with the least likelihood of human interference was selected. Accordingly, 60 camera trap placement sites were chosen (Fig 2); with the number of sampling points consistent with the first survey. The furthest spacing between scent-marking posts (3.13 km) fell within the chosen required maximum distance between camera trap placements (3.7km).

Cameras were set along the anticipated path of a passing cheetah to photograph the flank of the animal because broadside images facilitate easier identification [14]. Where possible, brush was packed around the scent-marking tree leaving only one access point to encourage the animals to move in front of the camera [14]. A combination of Cuddeback Attack (*n* = 24) and Bushnell Trophy Cam^TM^ IR (Bushnell Outdoor Products, Overland Park, Kansas, USA) (*n* = 6) camera traps were used. Cameras were only operational at 30 locations during any given sample occasion. Thus, the Adjacent Block method was implemented, with the sampled area divided into two blocks and a camera rotation after 45 consecutive days to cover the entire sampled area. Camera traps were set to take still images after a trigger event. However, to aid in the identification of captured individuals, the following settings were also implemented: The Bushnell cameras allowed a burst of three photographs per trigger, but for every trigger event, consecutive photographs were recorded as a single capture. Cuddeback Attack cameras allowed for a short video clip (30 seconds) to be taken after each daytime trigger event. This function was activated to aid individual cheetah identification. All other camera settings and positioning were as per the first camera trapping survey.

The survey ran for 90 days and was carried out during the cool/dry season (June–September 2013). Cameras were checked approximately every two weeks with an initial check after three days to ensure that the camera was operating correctly and was properly positioned to maximise the chances of captures.

To increase the number of captures, the survey period of the second camera trapping survey was extended after the initial 90 day survey. The 30 camera traps were left at their position for a further 40 days, extending the number of trapping days to a total of 130 days. While a long survey period may be necessary for species with low detectability to have sufficient captures for analyses [32], the assumption of demographic closure may become violated [32].

### Cheetah identification

Cheetah photographs were categorized and analysed with Adobe Photoshop Lightroom 3.6. All photographic captures of cheetahs were analysed by visual inspection of spot patterns to determine the identity of each cheetah and each individual was given a unique identity number [39, 40]. The identification of individuals and capture events was based on the guidelines below [40–43].

Individuals were identified based on spot patterns or individual spots on the body, tail, legs and face. At least two, but preferably three, unique features or human-made markers (e.g. a collar) were required to identify an individual. One different feature was considered sufficient to consider that two photographs represented two different individuals. Photographs of poor quality, or where spot patterns were obscured, were marked as unidentifiable and excluded from the analysis. A photograph was considered to be a first capture if it could not be matched with any individuals in previous photographs. Re-captures were photographs depicting an individual already identified. All individuals were sexed based on presence/absence of scrotal testes. Where the sex of an individual was not visible, it was categorized as unknown sex until a clearer photograph could be used to determine sex.

The photographs were independently analysed by two people to ensure their correct classification [44]. If an individual’s identity could not be agreed upon, these photographs were excluded from the analysis. A cheetah identikit, developed from photographic sightings, was also used to assist with identification [34]. Only adult cheetahs were considered for analysis of population estimates. The sampling occasion, time, location, and individual cheetah identity of each capture event were recorded in a spreadsheet. Capture histories were prepared for each adult identified in the camera-trapping survey.

### Data analysis

Tests for population closure were performed using the CloseTest program version 3. The program tests capture-recapture data for closure using two tests; the Stanley and Burnham [45] test and the Otis *et al*. [7] test. Density estimates were calculated in the program SPACECAP using information on capture histories in combination with the distribution of individuals (trap sites) and each traps’ active days (dates when camera trap locations were active and operational), providing more accurate, precise, and hence more reliable results [46]. SPACECAP uses a Bayesian modelling framework which offers non-asymptotic inferences which are applicable for small data samples typical of camera trapping studies of carnivores that occur at low densities [47]. SPACECAP also allows for inference about the locations of individuals that were not photographed during the survey and could thus be used for modelling a demographically open population [46]. The model firstly determines an individual’s activity centre and then estimates the density of these activity centres across a precisely defined area containing the trap array [47]. Furthermore, the models consider the traps as functioning independently and this allows individuals to be captured in multiple traps during a capture occasion and even multiple times by the same camera trap, which is realistic in camera-trapping studies [37].

SPACECAP runs as a package in the program R version 3.0.2 (R Development Core Team) [47]. SECR analysis in SPACECAP requires specific input files. Three input files are required; these files consist of the following:

1. Animal capture detail
2. Trap deployment detail
3. State-space detail

Guidelines for creating the three input files can be found in the SPACECAP manual [47].

The third file (state-space detail) requires the creation of potential activity centres within the state-space. The state-space or ‘S’ represents the surveyed area containing the camera traps combined with an extended area surrounding it. The state-space is represented by a fine grid of equally-spaced points that represent all possible activity centres (or home range centres) of all of the individuals in the population surveyed [47]. Point spacing of 1000 m was used in the point array. Potential home range centres were generated using ArcMap10 in conjunction with the Repeating Shapes for ArcGIS extension Tool [48]. The state-space requires being sufficiently large to ensure stability in the density estimate, which usually requires a buffer strip to be added to the trap array that is two or three times larger than the encounter probability parameter [47]. A “Minimum Area Rectangle” is formed by connecting the outermost camera trap locations in a rectangle and a buffer is created around this. The buffer region should be sufficiently large for individual animals outside the buffered region to have zero probability of being photo-captured by camera traps during the survey. For the analysis, the state-space boundaries were calculated using the Maximum Distance Moved (MDM) (Buffer width = 28 km) of a cheetah fitted with a satellite collar [34]. This was calculated by averaging all of the GPS co-ordinates. The furthest fix from the centre point of its home range was used to measure the MDM. The adequacy of the model was evaluated based on its Bayesian posterior probability (P-value). A model that provides an adequate description of the data will have a Bayesian P-value near 0.50; extreme values (near 1 or 0) indicate that the model is inadequate [47].

The habitat suitability indicator was created with data from Google Earth. Aerial imagery of the state-space area was used to indicate areas unsuitable for cheetahs. Selected by the authors, unsuitable areas included human settlements, large water bodies, fenced agricultural farms (farms with high human activity and maintained game fences), and mining areas [47, 49]. Home range centres that fell on these areas were identified as locations where cheetahs could not exist and marked with a ‘0’ next to their co-ordinates. Regions of suitable habitat were described by a grid of equally spaced points representing 1 km² over the state-space. The activity centres are assumed to be uniformly distributed over this area of suitable habitat.

The SPACECAP input files were uploaded and appropriate model combinations were chosen for analysis [47]. The following model definitions were selected: trap response absent, spatial capture-recapture, and detection function was set to half-normal [47]. The Markov-Chain Monte Carlo (MCMC) parameters were set to 200 000 iterations and burn-in values of 4000 generations to accommodate for the small sample size and large movements observed in the dataset, no thinning was selected (value of 1) and data augmentation of 35 was chosen. The data augmentation value represents the maximum allowable number of possible animals within the state-space [46]. The behavioural response was not chosen as baits or lures were not used in the survey, thus an individual’s encounter probability before and after the initial encounter was expected to be similar. Movement of individuals was non-random in this case as individuals will use certain scent-marking posts within their home range [41].

Density estimates for the two different sampling period lengths were compared using a Student’s t-test.

## Results

### Regular trap configuration

A total of 1616 active days were logged during which 3346 animal photographs were taken and only nine (0.27%) were photographs of cheetahs. Cheetah photographs were recorded at only two of the 60 sampling locations and all but one of these events occurred at a camera trap station that had been placed at a known cheetah scent-marking post. No further analyses to assess cheetah population size were carried out due to the insufficient number of cheetah captures.

### Non-random configuration using scent-marking posts

The second survey had a total of 2660 active camera trapping days and of the 3323 animal photographs, 53 (1.6%) were of cheetahs captured at 11 of the 60 camera trap sampling locations. Cheetah photographs made up 18 independent capture events. A total of seven adult cheetahs were identified from photographs (two females and five males) and five cheetah photographs were excluded from the analysis as the individuals could not be identified. However, the cheetahs in these photos were all cubs and would have been excluded from the analysis regardless. Details of individual cheetah visits, capture location and capture occasion are shown in S1 Appendix.

Capture frequency ranged from one to five per individual, with an average of 2.86 captures per individual. The number of photographs per sampling occasion ranged from one to nine, with an average of 2.79 per sampling occasion. Latency or time delay to first photograph for each individual ranged from 9 to 85 days.

A further 1090 days were logged from the extended survey period which resulted in a total of 3750 recorded active camera trapping days (Table 1). An additional seven camera trap locations photo-captured cheetahs, which accounted for a total of 13 events including 147 cheetah photographs, increasing the total sample size from 18 to 31 capture events at a total of 18 camera trapping locations (Table 1). No new individual cheetahs were recorded during this extended survey. However, capture frequency ranged from two to 10 per individual, with an average of 5.57 captures per individual. Capture details of individual cheetah visits are shown in S2 Appendix.

**Table 1:** Summary of the camera trapping surveys conducted in NOTUGRE.

	Regular trap configuration	Non-random configuration	Extended survey
No. of active camera-trapping days	1616	2660	3750
Total number of photo-captures	3346	3323	4823
Cheetah photo-captures	9	53	200
Cheetah capture events	5	18	31
Number of individual cheetah identified	2	7	7
Number of sampling locations that captured cheetahs	2	11	18
Capture frequency per individual (mean)	N/A	2.86	5.57

Closure tests for the second survey were inconclusive due to the small dataset. Small sample sizes and unequal capture probabilities can negatively affect closure tests [6, 7]. However, previous studies of large felids have indicated that a three-month sampling period is sufficient to meet the closure assumption [10, 42].

The SECR model estimated 0.61 ± 0.18 adult cheetahs per 100 km² for the 90-day period with a 95% maximum of 0.9 and minimum of 0.3 cheetahs/100km² (Table 2). An absolute abundance of four cheetahs (range: 2–6 individuals) was estimated for the ~700 km² reserve. However, NOTUGRE is probably too small to contain the home ranges of all resident cheetahs and it is therefore likely that the absolute abundance at any one time may be higher.

**Table 2:** Density estimates of cheetah using MDM buffer width of 28 km and MCMC parameters set at 200 000 iterations and 4000 burn-in generations. Density is expressed as the number of cheetahs per 100 square kilometres.

Variables	Mean	SD	95% Lower HPD level	95% Upper HPD Level	Bayesian posterior probability
Sigma	5.10	1.02	3.27	7.14	0.5
lam0	0.03	0.03	0.01	0.07	
Psi	0.67	0.20	0.32	1.00	
N_super_	28.56	8.38	14.00	42.00	
Density	**0.61**	0.18	0.30	0.90	

The population closure test for the extended survey period was also inconclusive due to insufficient data. The SECR population estimate for the 130-day period produced a density estimate of 0.58 ± 0.2 adult cheetahs/100 km² (abundance of four cheetahs; range 1–6 individuals). This estimate is slightly lower than the density estimated in the 90 day survey (0.61 ± 0.18 cheetahs/100 km²) (Table 2), but the population means did not differ significantly (t-test; *t* = 1.22; *df* = 226; p > 0.05). A summary of the extended survey are shown in Table 3.

**Table 3:** Density estimates calculated from capture histories of the extended survey using the MDM buffer width of 28 km and MCMC parameters set at 200 000 iterations and 4000 burn-in generations. Density is expressed as the number of cheetahs per 100 km².

Variables	Mean	SD	95% Lower HPD Level	95% Upper HPD Level	Bayesian P-value
Sigma	6.29	1.57	3.78	9.48	0.49
lam0	0.01	0.01	0.00	0.02	
Psi	0.64	0.22	0.25	1.00	
N_super_	27.10	9.23	11.00	42.00	
Density	**0.58**	0.20	0.24	0.90	

## Discussion

Typically, camera trapping surveys of large carnivores have camera trap stations set out systematically across the landscape (*sensu* our first survey) and camera traps are often placed along animal trails to maximize the probability that the target species is encountered and photographed. Animal paths channel animal movements and therefore normally provide ideal set up sites for camera traps. This systematic approach also ensures even coverage across the landscape and hence that every member of a population is likely to be included in the sample [10, 32]. However, the systematic approach is directed at species which travel on animal trails, and these species generally tend to be those species that occur in relatively densely vegetated environments. For example, the systematic grid approach of placing camera traps on trails has been used successfully to estimate the densities of tigers (*Panthera tigris*) [10, 42], jaguars (*Panthera onca*) [6, 12, 36, 50], ocelots (*Leopardus pardalis*) [51–53], leopards [11, 33, 54, 55] and pumas (*Puma concolor*) [44, 56], all of which occur in relatively closed habitats. In our first survey, cheetah capture was low with the only valid captures taken from a camera trap set at a known scent marking post. The low cheetah photo-capture rate and the small sample sizes are probably ultimately related to the low detection probability of cheetahs [5]. The probability of detection may have been affected by the choice of specific placement sites for cameras, habitat characteristics, sampling duration, density of the targeted species and its behaviour in the landscape [5]. Our study site is dominated by open landscapes and cheetahs do not seem to use animal paths preferentially. Imperfect detections are likely to be further heightened because our target species occurs at extremely low population densities.

The location and placement of camera traps is a critical component to a successful camera trapping survey [9, 57], and to a certain extent this component is overlooked in the regular trap configuration approach, particularly for species that do not necessarily choose to use animal paths. Careful choice of trap location may increase the probabilities of capturing the target species, and hence produce a more accurate representation of the true population at a site [6]. The design of the survey should have camera traps placed to maximize capture probability [9, 11]. Our second survey had camera traps located at targeted cheetah scent-marking posts identified by local guides.

Scent-marking posts provided ideal set up locations as they were frequently utilised by cheetahs [see also 14]. In addition to a higher probability of cheetah captures, cheetahs would stay at the scent-marking post long enough to obtain clear photographs; often sniffing the tree and scent-marking for a few minutes before moving on. This would not only give the camera the chance to capture the subject moving but also often resulted in multiple photos of an individual during a single capture event, sometimes providing a full individual profile (i.e. left- and right-hand side photographs; [14]). We note, however, that a possible drawback to using scent marking posts as camera trap locations is the possible variation in individual detectability, particularly in relation to age, sex and dominance [7]. It has been observed that female cheetahs may use scent-marking posts less frequently than males and that this difference in detection probability may bias estimates and under-estimate population abundance [3, 14, 29, 58]. Camera trapping at scent marking trees has further been cautioned as females rarely scent-mark unless they are in oestrous [59]. However, in our survey, two females were photo-captured at three scent-marking trees on three different occasions. These females were not believed to be in oestrous at the time. Although males use scent-marking posts more frequently than females [14, 60], provided that sufficient camera traps are used and that the study is carried out over a sufficiently long survey period, this bias should have a minimal effect on the results. Alternatively, where sample size permits, these sources of heterogeneity can be addressed by including sex-specific encounter rates. However, our survey did not have sufficient recaptures to allow such stratification [61]. Another important drawback of using targeted camera placement is irregular trap configuration and hence uneven sampling effort across the landscape. This shortcoming can be alleviated by choosing sites spread as evenly as possible across the surveyed area and accounting for habitat heterogeneity.

A capture-recapture study requires a relatively large number of recaptures to produce precise results [4, 7]. However, sample size is also affected by the size of the sampled area, the number of camera traps used, and the number of trapping occasions and, most importantly, on capture probability [7]. Sampling effort can be controlled through the size of the sampled area and the number of camera traps used [42]. It is traditionally recommended that the surveyed area be at least four times the size of the average home range of the target species [7], but this is logistically and financially impractical for a wide range of vertebrate species [32]. Alternatively, the duration of the survey can be extended judiciously. Ninety days is the recommended maximum number of days to maintain the population closure assumption when studying large felids [9]. However, when surveying species that occur at very low population densities, such as cheetahs [41], this recommended maximum number of trapping occasions may be insufficient due to the small sample size and high latency to first detection. Lengthening the sampling duration beyond this maximum may improve the robustness of the results but requires careful consideration of the temporal closure assumption [32]. Nonetheless, increasing the length of the survey may be appropriate for some species with long life expectancies and to areas with long seasons [62]. Furthermore, SPACECAP can calculate the density of demographically open populations [46]. Extending the total number of sampling occasions in our study provided substantially more photographs and independent captures, increasing the degree of certainty in the associated density [62]. However, no new individuals were captured and a larger sample size appeared to change the density estimate little, suggesting that the 90-day survey period provided sufficient data for a robust population estimate. However, the spatial scale of the study area (±240 km²) may have been insufficient to incorporate sufficient home ranges of all cheetahs, thereby rendering the population closure test inefficient. Our sample population must therefore be defined as open [7]. Finally, density results from within the reserve should not be used to extrapolate density outside the study area as there may be differences in vegetation cover, prey density, human activity, land-use, and in the density of other large carnivores. Furthermore, a potential limitation to our study is that cheetah detectability could well have changed over the course of the study. While we believe that this effect was likely very small in our study because of the low overall cheetah population, it should be considered in future research.

## Conclusion

Although it is important to have a standardised approach when designing a camera-trapping survey for large predator species, it is equally important to understand the limitations of individual surveys and adapt the method to report population size estimates as accurately as possible. However, the methods, and the justification for their use should be reported in detail so that the study can be adequately repeated and compared across different study populations.

Site specific targeted placement may be necessary for rare predator species that occur at low population densities and do not favour animal paths. This is particularly true for species which occur in more open landscapes, where animal paths are not necessarily used and the chances of movement in front of randomly set out camera traps is greatly reduced. Additionally, species with large home ranges may require extended survey period to accommodate for the high latency in detection. Nevertheless, the approach which we describe here could also be extended to non-felid carnivores. For example, brown hyenas (*Hyaena* brunnea) generally occur at low population densities and are notoriously erratic in their movements, but they make use of latrine sites [63]. Thus using site-specific, targeted placement of camera traps at latrine sites may present the most appropriate way to sample their populations, particularly in big open systems.

Providing precise estimate of abundance is key to understanding the status of a species, yet monitoring techniques for rare predator species that occur at extremely low densities are limited [3]. Camera trapping of rare species can be used to provide precise estimates of density but it is recommended that careful placement of camera traps is exercised, including the use of site specific targeted placement to increase the probability of detection in order to have reliable population estimates which are required to assess the viability of a population within an ecosystem.

## Supporting Information

S1 Appendix Detail of individual cheetah visits (sample occasions) recorded from the second camera trapping survey in the Northern Tuli Game Reserve, Botswana. Independent capture events used for analyses are not shown.
(DOCX)

S2 Appendix Capture details of individual cheetah visits (sample occasions) recorded during the extended second camera trapping survey in the Northern Tuli Game Reserve, Botswana. (DOCX)
