# Supplementary material for: Trapping Elusive Cats: Using Intensive Camera Trapping to Estimate the Density of a Rare African Felid
Source: PLoS One. 2015 Dec 23;10(12):e0142508. doi: 10.1371/journal.pone.0142508 (PMC4689357; doi:10.1371/journal.pone.0142508)
Supplement: S1 Appendix — Independent capture events used for analyses are not shown. (DOCX) [file pone.0142508.s001.docx]

| **Sample occasion*** | **Time** | **Location ID** | **Number of photographs** | **Cheetah ID** |
| --- | --- | --- | --- | --- |
| 9 | 11:46 | 24 | 1 | CM6 |
| 10 | 12:07 | 25 | 1 | CM5; CM6 |
| 38 | 10:49 | 25 | 1 | CM3 |
| 45 | 11:14 | 18 | 1 | CM2 |
| 46 | 14:31 | 22 | 2 | CM1; CM2 |
| 46 | 15:48 | 22 | 4 | CM1; CM2; unidentifiable |
| 50 | 13:20 | 58 | 3 | CF3; 2 cubs |
| 63 | 06:33 | 54 | 3 | CF3; 3 cubs |
| 72 | 06:33 | 56 | 5 | Cub |
| 78 | 05:18 | 33 | 3 | CM5; CM6; unidentifiable |
| 85 | 10:43 | 40 | 6 | CF4 |
| 87 | 19:34 | 58 | 2 | CM2; CM3 |
| 87 | 04:46 | 58 | 3 | CM1 |
| 87 | 04:51 | 58 | 2 | CM2 |
| 87 | 07:28 | 57 | 1 | CM1; CM2; CM3 |
| 87 | 07:45 | 56 | 2 | CM2; CM3 |
| 88 | 20:20 | 55 | 9 | CM1; CM2; CM3 |
| 88 | 05:09 | 55 | 1 | CM1 |
| 88 | 05:26 | 54 | 3 | CM1; CM2; CM3 |

*Sample occasion refers to the day on which cheetahs were captured within the survey period with sampling occasion 1 referring to the first day of sampling.
