# Supplementary material for: Trapping Elusive Cats: Using Intensive Camera Trapping to Estimate the Density of a Rare African Felid
Source: PLoS One. 2015 Dec 23;10(12):e0142508. doi: 10.1371/journal.pone.0142508 (PMC4689357; doi:10.1371/journal.pone.0142508)
Supplement: S2 Appendix — (DOCX) [file pone.0142508.s002.docx]

| Sample occasion | Time | Location ID | Number of photographs | Cheetah ID |
| --- | --- | --- | --- | --- |
| 101 | 1:58 | 33 | 2 | CM6 |
| 101 | 9:13 | 44 | 32 | CM5; CM6 |
| 124 | 11:41 | 51 | 1 | CF4 |
| 126 | 6:20 | 47 | 3 | CF8 (cub) |
| 130 | 2:37 | 52 | 3 | CM1; CM2; CM3 |
| 130 | 3:16 | 51 | 2 | CM2; unidentifiable |
| 130 | 3:36 | 33 | 2 | CM6; unidentifiable |
| 130 | 3:49 | 47 | 3 | CM2; unidentifiable |
| 130 | 7:43 | 41 | 9 | CM2; CM3 |
| 131 | 23:03 | 43 | 1 | CM5 |
| 131 | 3:42 | 38 | 3 | CM1; CM2; CM3 |
| 133 | 19:18 | 33 | 1 | CM5; CM6 |
